# Malnutrition and other risk factors of geriatric depression: a community-based comparative cross-sectional study in older adults in rural Bangladesh

**DOI:** 10.1186/s12877-021-02535-w

**Published:** 2021-10-18

**Authors:** Md. Ziaul Islam, Tasnim Rahman Disu, Sharmin Farjana, Mohammad Meshbahur Rahman

**Affiliations:** 1 Department of Community Medicine, National Institute of Preventive and Social Medicine (NIPSOM), Mohakhali, Dhaka, 1212 Bangladesh; 2 Institute of Public Health Nutrition (IPHN), Mohakhali, Dhaka, 1212 Bangladesh; 3 Department of Obstetrics and Gynecology, Shaheed Suhrawardy Medical College Hospital, Sher-E-Bangla Nagar, Dhaka, 1207 Bangladesh; 4 Department of Statistics, World University of Bangladesh, Dhaka, 1230 Bangladesh

**Keywords:** Geriatric depression, Malnutrition, GDS, MNA-SF, Older adults, Rural Bangladesh

## Abstract

**Background:**

Malnutrition and depression are highly prevalent in older adults and can lead to disparaging outcomes. Analytical studies on geriatric depression (GD) and its association with malnutrition are very scarce in Bangladesh, although the size of the older population is increasing fast in the country. The current study aimed to assess the association between malnutrition and depression and associated risk factors in rural older adults.

**Methods:**

A community-based comparative cross-sectional study was conducted in 600 older adult residents (aged ≥60 years) of three rural communities of Bangladesh from January to October 2019. The study enrolled two groups of participants; 300 depressed as cases and another 300 non-depressed older adults as a comparison group matching their age and living area. We used a semi-structured questionnaire to collect data through a face-to-face interview. Geriatric Depression Scale-15 was used to determine depression, and a score of ≥5 was considered as depressed. We used the Bangla version of the Mini-Nutritional Assessment-Short Form to assess nutritional status, which comprised questions related to appetite, weight loss, mobility, recent illness/stress, dementia/depression, and BMI, and considered a score of 0–7 as the cutoff score for malnutrition. Measures included baseline and personal characteristics, malnutrition, GD, and its associated risk factors. A binary logistic regression model was fitted to identify variables associated with the risk of GD.

**Results:**

The study found no significant difference in gender (male Vs. female) between depressed (44.0% Vs. 56.0%) and non-depressed (46.0% Vs. 54.0%) older individuals. The study revealed that malnutrition was significantly (*p < 0.01*) higher in depressed (56.0%) than in non-depressed (18.0%) rural older adults. The malnourished older adults had around three times (AOR = 3.155; 95% CI: 1.53–6.49, *p* = 0.002) more risk of having depression than the well-nourished older individuals. Older adults who were unemployed (AOR = 4.964; 95% CI: 2.361–10.440; *p* = 0.0001) and from lower and middle class (AOR = 3.654; 95% CI: 2.266–7.767; *p =* 0.001) were more likely to experience depression. Older adults having a ‘poor diet’ were more likely to experience depression (AOR = 3.384; 95% CI: 1.764–6.703; *p* = 0.0001). The rural older adults who were single (AOR = 2.368; 95% CI: 1.762–6.524; *p =* 0.001) and tobacco users (AOR = 2.332; 95% CI: 1.663–5.623; *p =* 0.003) were found more likely to experience depression.

**Conclusions:**

A significant association between malnutrition and depression was evident by the current study in the rural older individuals of Bangladesh. It will be a prolific initiative if policymakers merge malnutrition and the risk factors associated with geriatric depression in providing universal health care for better health and well-being of the rural older populations.

## Highlights

- A community-based comparative cross-sectional study enrolled 600 rural older adults
- Majority (56.0%) of the depressive rural older adults had malnutrition
- Risk of depression was higher (AOR = 3.115) in malnourished older adults
- Tobacco use was significantly associated with geriatric depression
- Employment, income, education, and poor diet were major risk factors of depression

## Background

The majority of the total population of Bangladesh resides in rural areas, and they still live below the poverty line [1]. Rural older people cannot meet their fundamental needs and access to healthcare facilities that may significantly increase the risk of malnutrition and geriatric depression (GD) [2, 3].

The older population of Bangladesh is susceptible to poor health as there are diverse risk factors encountered in providing good health. The household’s food insecurity, inadequate knowledge due to illiteracy, poor appetite, weight loss, lack of awareness predispose illnesses like malnutrition. Limited access of older adults to health facilities could be considered as another underlying cause that could lead to poor health and illness [4]. Besides these, the rural people confront an unhealthy environment, inadequate social supports, and poor health amenities, which intensify their health risks, cause malnutrition, and invite various communicable and non-communicable diseases, where the older people are worse victims [5, 6].

Some existing literature from the developing countries reported that the rural people suffered from different health risks due to poverty, unemployment, insufficient health facilities, and absence of social security, where the risk is severe in the older community [7–9]. A community-based study conducted among the population of Kalapara Upazila (one of our sampling areas) of Patuakhali district showed that for every 8000 people there is only one health facility [10]. These lacking; basic humanitarian needs, socio-economic insecurity, and limited access to health facilities create a burden of poor health among the rural older adults.

According to the World Health Organization (WHO), 80% of older people will be living in low- and middle-income countries in 2050. GD is a common health condition in rural communities of developing countries. Older people are constantly at threat of this health problem due to socio-economic, physical, and social environment, and factors related to aging [11–13]. Rural older people of the developing countries are the most vulnerable group for various physical and mental health consequences, and diverse age-specific sufferings [12–14]. Their quality of life deteriorates and needs comprehensive health care, and increases healthcare utilization costs [15, 16]. As a consequence, a remarkable segment of older adults suffers from major depression and commit suicide, although multiple other reasons can also be involved in most cases [17].

Several risk factors (i.e., biological, social, psychological, environmental, etc.) have been suggested in the development of GD such as being female, older, single or widow, smoker, drug user, and multiple medication users, and having lower educational status [18, 19]. Low income, being unemployed, financial insecurity, poor physical health like malnutrition, co-morbidities, sleeping disorders, frailty, loneliness, lack of social support, stressful life events, poverty, and cognitive impairment are also established as determinants of GD [20, 21]. Most of these determinants are identified through descriptive cross-sectional studies and association of risk factors with geriatric depression through analytical studies is very scarce. Comparative study based on two groups of rural older adult participants to find an association between GD and malnutrition is not evident in the remote costal context of a developing country like Bangladesh.

Malnutrition and depression are interrelated geriatric medical disorders and diverse studies revealed an interdependent relationship between these two geriatric health issues. Globally, the depression status of older adults has been well researched but still, it is an unfinished agenda [8]. A previous study conducted in Bangladesh examined the issue and reported a 24% prevalence of malnutrition among Bangladeshi hospital-attending older patients [22]. In developing a public-health response to GD and malnutrition, it is imperative to devise strategies, which may reinforce recovery, adaptation, and sound health in the older population. But, most of the developing countries confront challenges to ensure that their health and social systems are ready to address these major geriatric health issues.

Research has demonstrated that earlier diagnosis and treatment of malnutrition can lead to improved outcomes and better quality of life as well as reduced health consequences among older adults. However, studies especially community-based case-control studies concerning GD and malnutrition especially in a rural setting are scarce in Bangladesh. In this context, the present community-based case-control study investigated GD as the dependent variable while malnutrition and associated risk factors as independent variables among the rural older adults in Bangladesh. The study intended to explore the relationship between GD with malnutrition and identify risk factors associated with GD. The study findings could contribute to devising effective interventions and approaches to prevent these leading geriatric medical disorders in the country.

## Methods

### Aim, design, and settings of the study

**Fig. 1:**
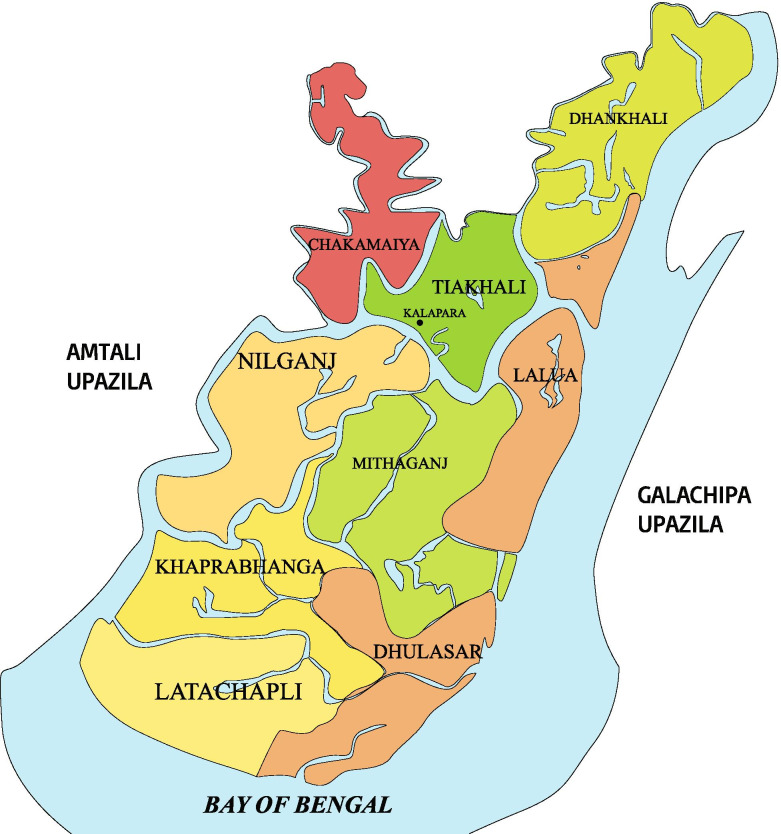
Study area with access to health facilities of southern rural region (Kalapara Upazila, Potuakhali District) of Bangladesh [10]

We conducted this community-based comparative cross-sectional study to assess the association between malnutrition and GD and ascertain the associated risk factors in rural older adults of Bangladesh. The study was conducted from January to October 2019 in three rural villages; Dhankhali, Tiakhali, and Lalua in Kalapara Upazila of Patuakhali district in Bangladesh. The villages are located in the remote coastal belt of the country bedsides the Bay of Bengal (Fig. 1).

### Participants

The study comprised two groups of participants; the depressed group included older adults (aged ≥60 years) having depression, and the non-depressed group included older adults having no depression. We identified the depressed and non-depressed older adults based on the score estimated by the Geriatric Depression Scale-15 (GDS-15). An older adult with a GDS score of < 5 was classified as non-depressed while an older adult with a score of ≥5 was classified as depressed. The study participants were included in the study having criteria such as; (i) a permanent resident of the selected rural community; (ii) aged ≥60 years; (iii) capable of giving informed written (or verbal) consent, and (iv) absence of severe illness (v) depressed older adults were regarded as cases while the non-depressed older individuals were regarded as controls for comparing the study findings.

### Sample size and sampling

Without considering the matching and considering 10% non-response rate, and rounding, a sample size of 600 older adults would allow identifying an odds ratio of 2 for exposure of 10.6% among controls (confidence level = 0.95 and power = 0.80). We recruited 300 older individuals having depression as a study group i.e. cases and another 300 individuals without depression as a comparison group i.e. controls who were individually matched for age (±2 years) and neighborhood (residence of depressed and non-depressed in the same rural community). We recruited 200 older individuals (100 depressed and 100 non-depressed) from each village and a total of 600 older individuals (300 depressed and 300 non-depressed) from three villages following the systematic random sampling technique. In the beginning, the principal investigator estimated the sampling interval using the total households and sample size in each of the three villages. Considering the sampling interval, the data enumerators attended the selected households for finding the older individuals living there. Based on the selection criteria, we identified the expected number of depressed (cases) and non-depressed (controls) older individuals for recruiting in the study.

### Data collection

A semi-structured questionnaire was applied for data collection by face-to-face interviews by trained interviewers of both sexes. The questionnaire was pretested among the older adults in a similar rural community of neighbor Upazila (sub-district) and finalized following necessary corrections and modifications based on the findings. All the data enumerators (interviewers) were medical graduates and had a Master of Public Health (MPH) degree. Interviews took place at the houses of both depressed and non-depressed older adults. The questionnaires consisted of a common set of questions, and information was obtained from both groups of older individuals. These included baseline and personal characteristics, malnutrition-related variables, and factors associated with depression. Before the interview, we took the informed consent from each older adult by explaining the objective and procedure of the study along with the risk and benefit of participation.

### Measures

#### Baseline characteristics

Information concerning baseline characteristics included age, gender, religion, marital status, monthly income, level of education, employment status, and family type. Although the country has four major religions, only two (i.e., Islam and Hinduism) were found in the present sample. Family income was used as an indicator of social class: less than 10,000 BDT (lower class), 10,000 to 20,000 BDT (middle class), and more than 20,000 BDT (upper class) based on the recommendations of Mamun MA et al., [23]. The marital status of being single included those who were divorced, separated, and widowed older adults.

#### Personal characteristics

Information concerning personal characteristics included psychosocial, physical health, lifestyle, and dietary factors. Relevant variables included peer group support history of suffering from chronic illnesses, active in daily life, having a poor diet, and tobacco use. For assessing peer group support, participants were asked if they received any type of support from friends and others. Regarding tobacco use, participants were asked about including smoking and smokeless tobacco use. Regarding active in daily life, participants were asked if they performed daily activities on their own such as shopping, household chores, washing clothes, etc. regarding having a poor diet, participants were asked if they had consumed the foods regularly, timely, adequately, and nutritious for health.

#### Geriatric depression

The depression in older adults was assessed with GDS-15, the most well-established scale for assessing GD, which has been validated and widely used in both community and clinical settings [24]. The GDS assessed depressive symptoms experienced in the preceding week. The scale comprises 15 questions requiring a binary (‘yes/no’) response. Out of the 15 items, 10 indicated the presence of depression when answered positively, while the other remaining (Items 1, 5, 7, 11, and 13) indicated depression when answered negatively. The scale has a total score of 15 (ranged 0 to 15), where depression level is classified into normal (scores 0–4) [25], and a score of ≥5 indicates probable depression [26]. The internal consistency of the scale in the present study was very good (Cronbach’s alpha = 0.85).

#### Malnutrition

The Bangla version of the Mini-Nutritional Assessment-Short Form (MNA-SF) was used for the collection of data on malnutrition. The revised MNA-SF of the MNA was developed and validated in Bangla especially for use among older adults [≥60 years] [27]. It comprised of six questions (relating to appetite, weight loss, mobility, recent illness/stress, dementia/depression, and BMI) and was scored from 0 to 14. A score of 0–7 was used as the cutoff score for malnutrition [28]. The internal consistency of this scale in the present study was very good (Cronbach’s alpha = 0.89).

### Statistical analysis

The data were analyzed using SPSS STATISTICS (Version 25.0, IBM Statistical Product and Service Solutions, Armonk, NY, USA) software. Descriptive statistics included frequency, percentage, means, and standard deviation. Inferential statistics considered a Chi-square test to assess the association between depression status and variables related to malnutrition, baseline, and personal characteristics of the older adults. All the variables statistically significant in bivariate analysis were entered into the binary logistic regression models [7]. The results of the logistic regression are reported as adjusted odds ratios with 95% confidence intervals (CI), and a *p*-value less than 0.05 was accepted as the significant level for this study.

## Results

A total of 600 rural older adults (300 depressed as cases and 300 non-depressed as controls) participated in this comparative cross-sectional study. The study found no significant differences in gender and age between cases and controls. Females shared 54.0% cases and 56.0% controls while males shared 46.0% cases and 44.0% controls. Regarding marital status, the majority of the cases (61.7%) were single, while the majority of the controls (76.0%) were married, and this difference was statistically significant (*p* < 0.01). In respect of social class (based on monthly family income), lower and middle class (having monthly family income ≤20,000 BDT) was significantly (*p* < 0.01) higher in cases (81.3%) than in controls (50.7%) while upper class (having monthly family income > 20,000 BDT) was significantly higher in the controls (49.3%) than in the cases (18.7%). In respect of educational status, higher education (Graduation and above) was significantly (*p* < 0.05) higher in the controls (56.4%)) than in the cases (43.6%). Concerning employment status, the majority of the cases (82.7%) were unemployed, while the majority of the controls (60.7%) were employed, and this difference was statistically significant (*p* < 0.01) (Table 1).

**Table 1:** Association between depression and baseline characteristics of older adults

Baseline Characteristic	Type of Participants		***p***-value
	Depressed (Case); n (%)	Non-depressed (Control); n (%)	
**Age group (Years)**			
60–69	154 (51.3)	198 (59.3)	0.233
70–79	86 (28.7)	82 (27.3)	
≥80	60 (20.0)	40 (13.3)	
Total	300 (100.0)	300 (100.0)	
**Gender**			
Male	138 (46.0)	132 (44.0)	0.481
Female	162 (54.0)	168 (56.0)	
Total	300 (100.0)	300 (100.0)	
**Religion**			
Islam	263 (88.0)	276 (92.0)	0.248
Hinduism	37 (12.0)	24 (8.0)	
Total	300 (100.0)	300 (100.0)	
**Marital Status**			
Single	184 (61.3)	72 (24.0)	0.001
Married	116 (38.7)	228 (76.0)	
Total	300 (100.0)	300 (100.0)	
**Social Class (Based on monthly family income)**			
Upper class (> 20,000 BDT)	56 (18.7)	148 (49.3)	0.001
Lower and Middle class (≤20,000 BDT)	244 (81.3)	152 (50.7)	
Total	300 (100.0)	300 (100.0)	
**Educational status**			
Illiterate	62 (20.7)	30 (10.0)	0.026
Non-institutional education	52 (17.3)	68 (22.7)	
Between class 1–5	44 (14.7)	38 (12.7)	
Between class 6–10	54 (18.0)	32 (10.7)	
S.S.C	18 (6.0)	28 (9.3)	
H.S.C	36 (12.0)	60 (20.0)	
Graduation and above	34 (43.6)	44 (56.4)	
Total	300 (100.0)	300 (100.0)	
**Employment Status**			
Employed	52 (17.3)	182 (60.7)	0.001
Unemployed	248 (82.7)	118 (39.3)	
Total	300 (100.0)	300 (100.0)	
**Family Type**			
Nuclear family	224 (74.7)	228 (76.0)	0.789
Joint family	76 (25.3)	72 (24.0)	
Total	300 (100.0)	300 (100.0)	

*n* number of observations; *%* Percentage; *S.S.C.* Secondary School Certificate; *H.S.C.* Higher.

Secondary School Certificate; Significance: Chi-square test, *p* < 0.05 with 95% CI.

Regarding the association between GD with selected personal characteristics, Pearson’s chi-square test showed that the depressed (cases) had significantly (*p* < 0.05) less peer group support than the non-depressed (controls) older adults (50.7% Vs. 64.0%). Active daily life was also significantly (*p* < 0.01) less prevalent in the cases than the controls (46.7% Vs. 64.7%). Having a poor diet was significantly (*p* < 0.01) higher in the cases than in the controls (37.3% Vs. 77.3%). Tobacco use (both smoking and smokeless tobacco) was significantly (*p* < 0.01) higher in the cases than in the controls (55.7% Vs. 37.3%). The study didn’t find any significant differences in chronic diseases between cases and controls (Table 2).

**Table 2:** Association between depression and personal characteristics of older adults

Personal characteristic	Type of Participants		***p***-value (Chi-square test)
	Depressed (Case); n (%)	Non-depressed (Control); n (%)	
**History of chronic disease**			
Having history	176 (58.7)	146 (45.3)	0.132
No History	124 (41.3)	154 (54.7)	
Total	300 (100.0)	300 (100.0)	
**Peer group support**			
Yes	152 (50.7)	192 (64.0)	0.020
No	148 (49.3)	108 (36.0)	
Total	300 (100.0)	300 (100.0)	
**Active in daily life**			
Yes	140 (46.7)	194 (64.7)	0.002
No	160 (53.3)	106 (35.3)	
Total	300 (100.0)	300 (100.0)	
**Tobacco use (Smoking and SLT)**			
Yes	167 (55.7)	112 (37.3)	0.003
No	133 (44.3)	188 (62.7)	
Total	300 (100.0)	300 (100.0)	
**Having a poor diet**			
Yes	188 (62.7)	68 (22.7)	0.001
No	112 (37.3)	232 (77.3)	
Total	300 (100.0)	300 (100.0)	

*n* number; *%:*Percent; Significance, *p* < 0.05 with 95% CI; *SLT* Smokeless tobacco.

The study found a significant (*p* < 0.01) association between malnutrition and GD, where 56.0% of the cases and 18.0% of the controls had malnutrition. The study also found that 82.0% of the controls and 34.0% of the cases had no malnutrition (Table 3).

**Table 3:** Association between depression and malnutrition in rural older adults

Malnutrition	Type of participants		***p***-value
	Depressed (Case); n (%)	Non-depressed (Control); n (%)	
Yes	168 (56.0)	54 (18.0)	0.001
No	132 (44.0)	246 (82.0)	
**Total**	**300 (100.0)**	**300 (100.0)**	

*n* number of observations; *%* Percent; Significance: Pearson’s Chi-square test, *p* < 0.05.

The logistic regression analysis showed that single older adults were more likely to experience depression (AOR = 2.368; 95% CI: 1.762–6.524; *p =* 0.001). Concerning social class, the lower and middles classes were more likely to experience depression (AOR = 3.654; 95% CI: 2.266–7.767; *p =* 0.001). The malnourished rural older adults had more than three times higher [AOR = 3.155, 95% CI: 1.534–6.494, *p* = 0.002] risk of having depression than the well-nourished older adults. The unemployed older adults had around five times higher (AOR = 4.964; 95% CI: 2.361–10.440; *p =* 0.001) risk of having depression, and older individuals having a poor diet had more than three times higher (AOR = 3.384; 95% CI: 1.764–6.703; *p* = 0.001) risks of having depression. The rural older adults who were tobacco users had more than two times higher risk of having depression (AOR = 2.332; 95% CI: 1.663–5.623; *p =* 0.003). The study also found a lower risk of depression among the older adults having SSC or below education than those having HSC or above education (Table 4).

**Table 4:** Logistic regression analysis of the factors associated with depression in older adults

Associated factor	Adjusted Model		
	Adjusted odds ratio (AOR)	95% Confidence Interval (CI)	***p***-value
**Marital status**			
Married	Reference		0.001
Single	2.368	(1.762–6.524)	
**Social class (Based on monthly family income)**			
Upper class (> 20,000 BDT)	Reference		0.001
Lower and Middle class (≤20,000 BDT)	3.654	(2.266–7.767)	
**Educational status**			
H.S.C and above	Reference		0.023
S.S.C and below	0.730	(0.556–0.957)	
**Employment status**			
Employed	Reference		0.001
Unemployed	4.964	(2.361–10.440)	
**Peer group support**			
Yes	Reference		0.935
No	0.947	(0.484–1.807)	
**Active in daily life**			
Yes	Reference		0.266
No	1.465	(0.748–2.871)	
**Having a poor diet**			
Yes	3.438	(1.764–6.703)	0.001
No	Reference		
**Tobacco use (Both smoking and SLT)**			
Yes	2.332	(1.663–5.623)	0.003
No	Reference		
**Malnutrition status**			
Well-nourished	Reference		0.002
Mal-nourished	3.155	(1.534–6.491)	

*SLT* Smokeless tobacco; *S.S.C.* Secondary School Certificate; *H.S.C.* Higher Secondary School Certificate; *AOR* Adjusted odds ratio; *CI* Confidence interval; Significance: *p* < 0.05 with 95% CI.

## Discussion

Diverse literature depicts that the causal relationship between malnutrition and depression in older adults is still inconclusive. Both malnutrition and depression are inherently related; depression may lead to appetite loss and undernutrition, while malnutrition may denigrate depression and apathy [29]. We conducted this comparative or analytical cross-sectional study in the rural setting of Bangladesh to assess the association between malnutrition and geriatric depression and identify associated risk factors. A study reported that older depressed people have a greater risk of psychological disorders, which provoke suicide and suicidal behaviors [30]. The existing health care delivery system in Bangladesh provides limited health care for older adults in urban communities, but it is scarce in rural settings. Accordingly, the rural older individuals do not get need-based health services within the rural health facilities. Robust data on these health issues and related risk factors are essential for devising an effective solution to reduce the occurrences of depression and malnutrition in the rural older population of the country.

A comprehensive literature review revealed that relevant data on GD in association with malnutrition is not available in the context of rural Bangladesh. Consequently, the present comparative cross-sectional study investigated malnutrition concerning GD and associated risk factors in rural older adults of the country. The current study is an ingenious initiative using a community-based analytical cross-sectional study design to expose the picture of geriatric depression and malnutrition in the rural context of the country. The study conserves enormous academic and policy implications because the study attempted to determine the temporality of the association between malnutrition and geriatric depression in the rural setting.

The present study showed that majority of the depressed and non-depressed (51.3 and 59.3%) older adults were aged 60–69 years. Another study conducted among the rural residents of Narail Upazila of Bangladesh reported a similar finding where 58.7% of those aged 60–69 years were suffering from depression as a psychological disorder [31]. The current study found that single older adults, lower education, unemployment, and less monthly income were significantly associated with GD. In the context of Bangladesh, the rural older adults are mostly kept unemployed and dependent on other family members, which compel them to suffer from financial scarcity, lack of access to nutritious foods, healthy living, and need-based health care. A study conducted by Haseen et al. reported that illiteracy and unemployment aggravated economic constraints and poverty, which sequentially contributed to malnutrition and the occurrence of geriatric depression [32].

The current study portrayed that majority (50.7%) of the cases (depressed rural older adults) had significantly (*p* < 0.05) less peer group support than the controls (64%). Another study conducted by Disu TR and colleagues found that 31.3% of the older individuals having peer group support were depressed (cases in the present study), and 68.7% were non-depressed (controls in the present study) [33]. In the local context of Bangladesh, rural older adults have poor access to peer group support due to the prevailing social structure, community design, and lifestyles of local people. The rural older people of the country confront these contextual realities and suffer from loneliness, mental stress, and depression.

The present study revealed that poor-diet consumption was significantly (*p* < 0.01) higher in depressed (62.7%) than in non-depressed (22.7%) older adults. The study depicted that the rural older individuals having a poor diet had more than three times higher risk of experiencing depression. Another study conducted by Disu TR et al. found that 77% of the older persons having a poor diet were depressed (cases in the present study), and 22.6% were non-depressed (controls in the present study) [33]. This difference could be due to the variation in methodological factors such as the sample size (small vs. large), sampling (systematic random vs. convenience), study design (comparative cross-sectional vs. descriptive cross-sectional), and study place (Rural vs. urban and rural both) between the current and former studies. It is also evident that ‘poor diet’ intake invites malnutrition in rural older populations.

Tobacco use was significantly (*p* < 0.01) higher in the depressed older adults (55.7%) than in the non-depressed (37.3%) older individuals. The study revealed that older people who were tobacco users had more than two times higher risk of experiencing depression. The research conducted by Disu et al. didn’t find any significant difference, where 39.3% of the older adults who were smokers had depression [33]. It could be claimed that our study assessed tobacco use considering both smoking and smokeless tobacco use while the other study considered smoking only. Moreover, variations in study design and places could also contribute to the differences. It is observed that rural older males use to do smoking in the form of bidi, cigarettes, hookah, and pipes. On the other hand, the rural older females use to take smokeless tobacco in the form of *Jarda, Gul, Sadapata, Nasshi, Khaini,* etc. [34]. Concerning the relation of depression with tobacco use, it is evident that tobacco stimulates the dopamine section from the pituitary gland of the human brain, which stimulates systemic functions and gradually produces neurological disorders like depression among older adult tobacco users.

The current study found that older adults engaged were engaged in sweat-producing activities like engagement in daily household activities and regular exercise had lower levels of depression. Some previous relevant studies also depicted similar observations. Depression is associated with decreased energy, increased fatigue, loss of interest in daily activities, and less concentration on daily activities [35, 36]. Our study found that the depressed rural older adults (46.7%) were significantly less active in their everyday life in compassion to the counterpart non-depressed older individuals (64.7%). In this regard, the study of Disu and colleagues found that 29.2% depressed and 70.8% non-depressed older people were active in daily life [33]. These differences could be due to variations in study place and population. It could be explained by the fact that active involvement in such activities mediates the release of various bodily chemicals like endorphins, norepinephrine, serotonin, etc., which help to prevent the occurrence of depression in older adults [37].

Our study revealed that more than half of the depressed rural older adults had malnutrition, while it was only 18% in the non-depressed older individuals. The malnourished older adults had more than three times higher risk of having depression. In this concern, another study depicted that depression is significantly affected by malnutrition, which is associated with the food-intake behavior of older adults [38]. The research carried out by German L and colleagues also found malnutrition remarkably higher in depressed than in non-depressed patients [39]. Since malnutrition has a significant influence on appetite and eating habits, it seems that having malnutrition could be a risk factor for older people to be depressed. Furthermore, food insecurity, non-availability, and inaccessibility might have an association with depression [38], particularly in the older adults [40], and taking a poor diet may lead to mood disorders, depression, and poor cognitive performance among this vulnerable group [41].

Given that rural older adults are more likely to live in an extended family with financial scarcity, poor diet, poor social support, and poor access to health care. As a result, they are more vulnerable to malnutrition, consequently suffer from mental stress and depression. Furthermore, studies indicate that diverse food elements like micronutrients, trace elements, and vitamins are also associated with the occurrence of different neurological complications like degenerative changes of the brain in late life, which could lead to depression [42, 43].

### Strength and limitations

Use of self-report and the non-representative sample from three selected rural communities of a district are few limitations to the generalizability of findings. Those who said they had a current acute illness (excluding long-term chronic conditions) were not eligible to participate in the study. Therefore, the prevalence of malnutrition was likely to be lower in both cases and controls. The study also missed some relevant risk factors like chronic diseases, comorbidities, and psycho-social factors that may affect GD. Despite these limitations, the present community-based case-control study identified crucial risk factors associated with depression in the rural context of the country where relevant data are scarce. The study findings also preserve essential policy inferences in devising effective interventions and health programs to prevent these leading geriatric health disorders in the rural older population of Bangladesh and other developing countries.

## Conclusion

The current study intended to identify the temporality of the relationship between malnutrition and GD. The study suggested a wide range of risk factors, where there is a lack of relevant data in the rural context of the country. The study findings would contribute to devising specific programs for improving the wellbeing of rural older adults. The study proposes diverse interventions like mental health education, awareness-raising programs, and nutritional supplementations to reduce the burden of malnutrition and GD in the rural older population.

## Data Availability

Availability was only given to the participants for their information and specific members of the study. The datasets generated and/or analysed during the current study are not publicly available as the ethical vote did not include open data access but are available from the corresponding author on reasonable request.

## Abbreviations

AOR: Adjusted odds ratio
CI: Confidence interval
GD: Geriatric depression
GDS-15: Geriatric depression scale-15
HSC: Higher secondary school certificate
IPHN: Institute of public health nutrition
IRB: Institutional review board
MNA-SF: Mini-nutritional assessment-short form
MPH: Master of public health
NIPSOM: National institute of preventive and social medicine 20
SPSS: Statistical package for social science
SSC: Secondary school certificate
TK: Taka (currency of Bangladesh)
WHO: World health organization
